# Supplementary material for: The interactome of intact mitochondria by cross-linking mass spectrometry provides evidence for coexisting respiratory supercomplexes
Source: Mol Cell Proteomics. 2017 Dec 8;17(2):216–32. doi: 10.1074/mcp.RA117.000470 (PMC5795388; doi:10.1074/mcp.RA117.000470)
Supplement: Supplemental Data [file supp_RA117.000470_133922_0_supp_23978_fzffwf.zip › spectra_annotation/mito_DR_spectra_annotation/328-1-3-1-5-1.pdf]

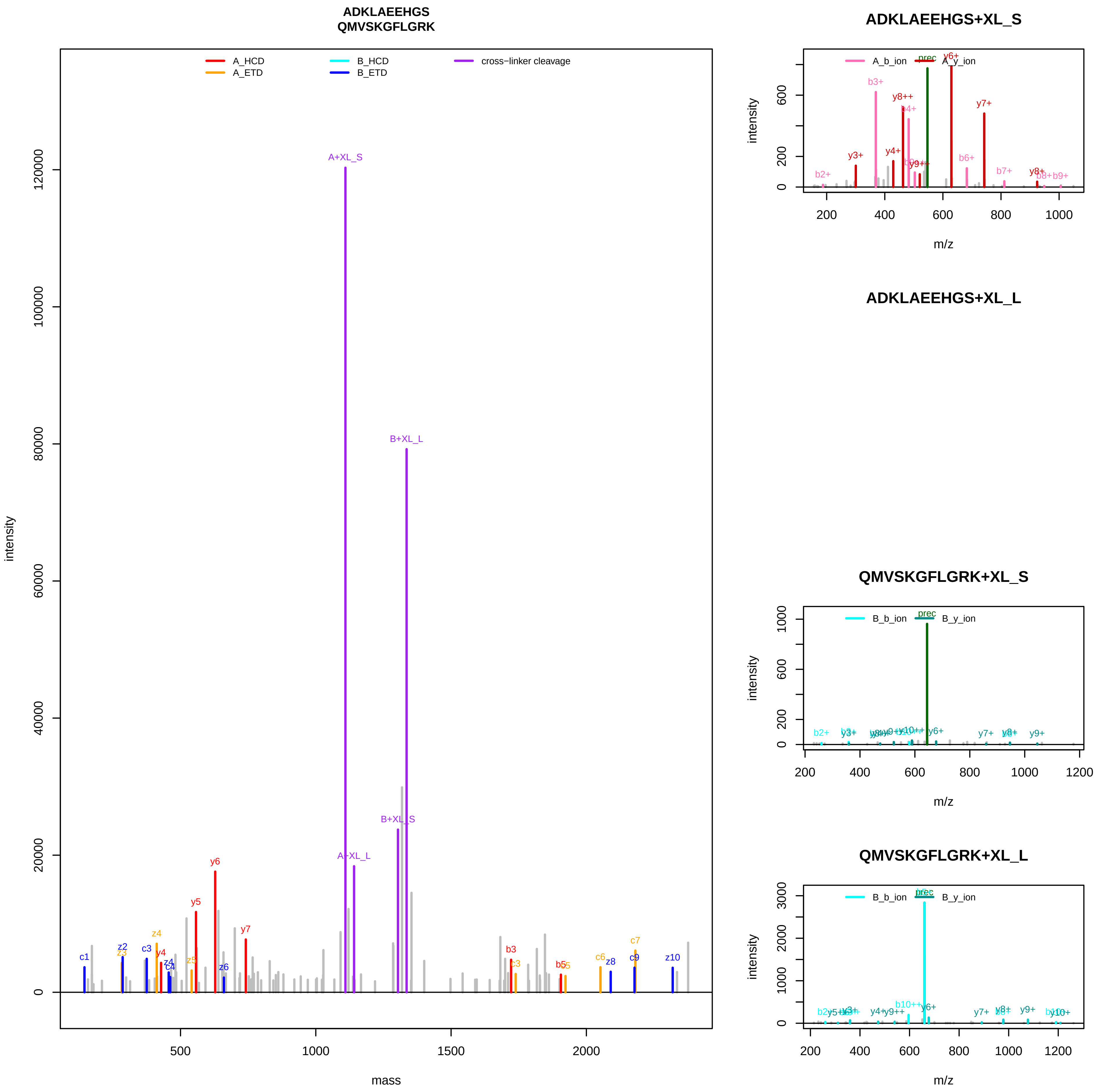
ADKLAEEHGS
QMVSKGFLGRK
A_HCD
A_ETD
B_HCD
B_ETD
cross−linker cleavage
A+XL_S
B+XL_L
B+XL_S
A+XL_L
intensity
mass
y5
y6
y7
y4
z4
z3
z2
c1
c3
c4
z5
z6
b3
c3
b5
c5
c6
z8
c9
c7
z10
ADKLAEEHGS+XL_S
A_b_ion
A_y_ion
prec
b3+
y8++
b4+
y6+
y7+
y3+
y4+
b6+
b7+
b2+
y8+
b8+
b9+
m/z
ADKLAEEHGS+XL_L
QMVSKGFLGRK+XL_S
B_b_ion
B_y_ion
b2+
y3+
y6+
y7+
y8+
y9+
QMVSKGFLGRK+XL_L
b10++
y4+
y9++
y10+
